# Stem cells and degenerative diseases

**DOI:** 10.1186/1753-6561-8-S4-O19

**Published:** 2014-10-01

**Authors:** Antonio Carlos de Carvalho

**Affiliations:** 1 Instituto de Biofisica Carlos Chagas Filho, Rio de Janeiro, Brazil

## 

Cell therapies utilizing stem cells hold a great promise for the treatment of chronic-degenerative diseases. For more than a decade now, pre-clinical and clinical studies have been using bone marrow derived cells for therapy of diverse degenerative diseases. Cardiologists have unarguably performed the largest number of clinical trials and we will concentrate our presentation on the use of various cell therapies in heart diseases. The first wave of clinical trials using bone marrow derived mononuclear cells is close to an end and results are rather disappointing. Most controlled studies, using multicenter, double-blinded and placebo-controlled designs have not shown heart function improvement (based on measurements of ejection fraction - EF% - as a surrogate endpoint). This has been observed for acute and chronic myocardial infarction [1-3], Chagasic cardiomyopathy [4] and more recently for dilated cardiomyopathy [unpublished results]. Clinical trials using mesenchymal stem cells, derived either from bone marrow or adipose tissue are underway [5]. These cells have been proofed safe but efficacy trials are still underway. Similarly, trials using cardiac derived stem/progenitor cells have been initiated with either c-kit positive [6] or cardiosphere derived cells [7]. Safety trials have been concluded and point to significant reduction in scar area in infarcted patients. Efficacy trials are currently starting. Pluripotent cells, either embryonic stem cells (ESC) or induced pluripotent stem cells (iPSC), have been tested in pre-clinical models of acute and chronic myocardial infarction with reported success [8], although permanent engraftment of these cells has been questioned. Finally, directed differentiation of fibroblasts into cardiomyocytes, using transcription factors, has been accomplished both in vitro and in vivo [9,10], opening new therapeutic avenues for handling the epidemic of heart failure that has followed improved survival after myocardial infarction.
